# Unveiling a hotspot of genetic diversity in southern Italy for the endangered Hermann’s tortoise *Testudo hermanni*

**DOI:** 10.1186/s12862-022-02075-w

**Published:** 2022-11-07

**Authors:** Andrea Chiocchio, Mauro Zampiglia, Marta Biaggini, Roberto Biello, Luciano Di Tizio, Francesco Luigi Leonetti, Oliviero Olivieri, Emilio Sperone, Massimo Trabalza-Marinucci, Claudia Corti, Daniele Canestrelli

**Affiliations:** 1 Dipartimento di Scienze Ecologiche e Biologiche, Università degli Studi della Tuscia, Largo dell’Università s.n.c, 01100 15 Viterbo, Italy; 2 Laboratorio Centrale per la Banca Dati Nazionale del DNA, Dipartimento dell’Amministrazione Penitenziaria, Ministero della Giustizia, via del Casale di San Basilio 168, Rome, Italy; 3 Museo “La Specola”, Museo di Storia Naturale dell’Università degli Studi di Firenze, Via Romana 17, 50125 Firenze, Italy; 4 Dipartimento di Scienze della Vita e Biotecnologie, Università degli Studi di Ferrara, Via Luigi Borsari 46, 44121, 13 Ferrara, Italy; 5 Societas Herpetologica Italica, Sezione Abruzzo-Molise, Via Federico Salomone 112, 66100, Chieti, Italy; 6 DiBEST, Università della Calabria, via P. Bucci, 87036, Rende, CS Italy; 7 Dipartimento di Medicina Veterinaria, Università degli Studi di Perugia, Via San Costanzo 4, 06126, Perugia, Italy

**Keywords:** Biodiversity hotspots, Conservation genetics, Italian Peninsula, Genetic structure, Phylogeography, Threatened species

## Abstract

**Background:**

Hotspots of intraspecific genetic diversity represent invaluable resources for species to cope with environmental changes, and their identification is increasingly recognized as a major goal of conservation ecology research. However, even for iconic and endangered species, conservation strategies are often planned without thorough information on the geographic patterns of genetic variation. Here, we investigated the spatial patterns of genetic variation of the endangered Hermann’s tortoise *Testudo hermanni* in the Italian Peninsula by genotyping 174 individuals at 7 microsatellite loci, with the aim to contribute to planning effective conservation strategies.

**Results:**

Ordination-based and Bayesian clustering analyses consistently identified three main genetic clusters, one spread in the central and northern part of the peninsula, and two restricted to southern Italy and Sicily, respectively. The highest levels of genetic diversity were found in populations of the southern cluster and, in particular, at the northern edges of its distribution (He > 0.6, Ar > 2.8 ), that correspond to areas of putative secondary contact and admixture between distinct lineages. Our results clearly identify a hotspot of genetic diversity for the Hermann’s tortoise in southern Italy.

**Conclusion:**

We inferred the evolutionary history and the spatial patterns of genetic variation of the Hermann’s tortoise in the Italian Peninsula. We identified three main genetic clusters along the peninsula and a hotspot of intraspecific diversity in southern Italy. Our results underline the urgent need for conservation actions to warrant the long-term persistence of viable tortoise populations in this area. Furthrmore, these data add further evidence to the role of southern Italy as a biodiversity hotspot for temperate fauna, claiming for higher consideration of this area in large scale conservation programs.

**Supplementary Information:**

The online version contains supplementary material available at 10.1186/s12862-022-02075-w.

## Introduction

Hotspots of genetic diversity are geographic areas harbouring exceptionally high levels of intraspecific genetic variation and represent appealing resources to investigate the mechanisms shaping the geographic structure of biodiversity [1–3]. Still, as genetic variation provides populations with the potential to adapt to environmental changes [4, 5], hotspots of genetic diversity also represent invaluable resources for species to cope with the global change [3, 6]. Thus, the identification of hotspots is a primary goal of evolutionary research, as well as a crucial step for designing effective conservation strategies for the long-term persistence of species and populations [7–10].

In the western Palaearctic region, Mediterranean peninsulas have been identified as major hotspots of intraspecific genetic variation for temperate species [11], and references therein]. The concentration of hotspots in these areas has been linked to the outcomes of Pleistocene glacial cycles, which induced populations to repeated cycles of fragmentation and allopatric differentiation, followed by secondary contact and admixture [2, 11–14]. However, during the last decade a new generation of phylogeographic studies has outlined more complex patterns of intraspecific genetic structure within Mediterranean peninsulas. In particular, two prominent patterns have been recurrently observed: (i) the occurrence of multiple, distinct evolutionary lineages within each single peninsula [15–21], and (ii) remarkable variation in the levels of genetic diversity among populations [14, 22–25]. Still, several studies have shown that the spatial resolution of a hotspot is smaller than previously believed, as in these areas the species genetic structure can be very scattered [10, 20]. Therefore, the single peninsula cannot be treated as a unique, homogenous hotspot, especially in a conservation genetic perspective, where the proper definition of evolutionary and management units is mandatory to successfully define conservation plannings [26]. This evidence stresses the need for more detailed data on the geographic structure of genetic variation of threatened/endangered taxa, in order to set proper conservation programs accounting for population evolutionary history and genetic diversity levels.

In this study, we focus on the geographic patterns of genetic variation of an endangered tortoise species, the Hermann’s tortoise *Testudo hermanni* Gmelin, 1789. The Hermann’s tortoise is a land tortoise inhabiting coastal and sub-mountain regions of the Italian and Balkan peninsulas, some Mediterranean islands, and two small areas of eastern Spain and southern France [27]. Hermann’s tortoise populations have declined markedly in the last 40 years, mainly due to coastal habitat destruction (including fires) and overharvesting for pet-trades [28–30]. Genetic investigations on mitochondrial markers identified two main genetic clusters, corresponding to the two recognized subspecies: *T. h. hermanni*, which includes populations from the Italian Peninsula, the western Mediterranean islands, and the isolated Spanish and French populations; *T. h. boettgeri*, which includes populations from the Balkan Peninsula and northern Italy [31]. Further genetic sub-structuring has been recognized between the eastern and the western Balkan populations, between the Italian and the western Mediterranean populations - although western Mediterranean populations were considered as introduced by humans during the Neolithic - and among populations within the Italian Peninsula [32]. However, previous studies did not analyse population structure and genetic diversity in southern Italy, a region usually harbouring multiple evolutionary lineages and non-negligible levels of diversity in many terrestrial vertebrates [14, 18, 20]. Interestingly, a recent study aimed at identifying the geographic origin of confiscated tortoises using microsatellite loci revealed some level of genetic differentiation in Hermann’s tortoise individuals from southern Italy [30, 33].

Here we employed a set of seven microsatellite loci to analyse the fine-scale population genetic structure of the Hermann’s tortoise in the Italian Peninsula, with a particular effort on the southernmost part of its range. We aimed to (i) providing further insights on the Hermann’s tortoise genetic structure and evolutionary history within the Italian Peninsula, (ii) identifying its hotspot of genetic diversity, (iii) mapping the geographic distribution of its evolutionary lineages. Considering the ongoing reduction of the Hermann’s tortoise habitat, this information will provide fundamental new insights to define effective strategies for the conservation of its populations.

## Results

The dataset consisted of a multi-locus genotype for 174 wild Hermann’s tortoise individuals (see Table 1) at seven microsatellite loci, with 10.3% of missing data (available on ZENODO repository following the link 10.5281/zenodo.6566199). The inspection of null allele occurrences with Micro-Checker [34] revealed the possible null alleles at locus *Gal236* in populations 8, 11, 13 and 14, and at Test10 in populations 3. However, no significant deviation from the Hardy-Weinberg and linkage equilibria was found after the Bonferroni correction was applied in FSTAT [35].

**Table 1:** Sample number, collecting locality, geographic coordinates, sample size (analysed specimens), allelic richness (Ar) and expected heterozygosity (He)

Sample	Location	Latitude	Longitude	Size	Ar	He
1	Vernazza	44.14	9.70	1		
2	Camaiore	43.92	10.30	3	2.571	0.543
3	Pian di Rocca	42.81	10.83	10	2.134	0.497
4	Uccellina	42.64	11.10	2		
5	Viterbo	42.42	12.13	1		
6	Manziana-Tolfa	42.15	12.06	8	2.472	0.574
7	Macchia Grande	41.82	12.22	7	2.392	0.549
8	Castel Porziano	41.71	12.40	9	2.38	0.555
9	Sabaudia	41.34	13.07	4	2.355	0.5
10	Baia Domizia	41.22	13.77	6	2.036	0.396
11	Torino di Sangro	42.22	14.56	20	2.354	0.514
12	Campomarino	41.92	15.10	4	2.156	0.541
13	Larino	41.85	14.88	5	2.117	0.438
14	Lesina	41.89	15.37	17	2.381	0.532
15	Conza	40.85	15.33	1		
16	Trasanello	40.67	16.67	1		
17	Laterza	40.62	16.78	3	2.143	0.49
18	Taranto	40.65	17.18	1		
19	Tarsia	39.62	16.26	2		
20	Roggiano Gravina	39.62	16.17	3	3	0.648
21	San Marco Argentano	39.56	16.12	8	2.456	0.538
22	Campana	39.41	16.83	4	2.628	0.566
23	Santa Rania	39.18	16.79	9	2.838	0.615
24	Roccabernarda	39.13	16.88	7	2.935	0.647
25	Simeri Crichi	38.95	16.66	11	2.439	0.491
26	Curinga	38.82	16.27	4	2.301	0.449
27	San Luca	38.16	15.97	3	2.171	0.419
28	Nebrodi	37.93	14.58	12	2.64	0.541
29	Castel di Judica	37.50	14.71	1		
30	Caltagirone	37.13	14.52	6	2.438	0.526
31	Caltanissetta	37.53	14.12	1		

Allelic richness and the unbiased mean expected heterozygosity estimated for each population using the *adegenet* R package [36] are shown in Table 1. Population 20 (Roggiano Gravina), 23 (Santa Rania) and 24 (Roccabernarda) from the north-central Calabrian region showed the highest values of genetic diversity (both heterozygosity and allelic richness), whereas the lowest values of genetic diversity were observed in population 10 (Baia Domizia, central Italy).

**Fig. 1:**
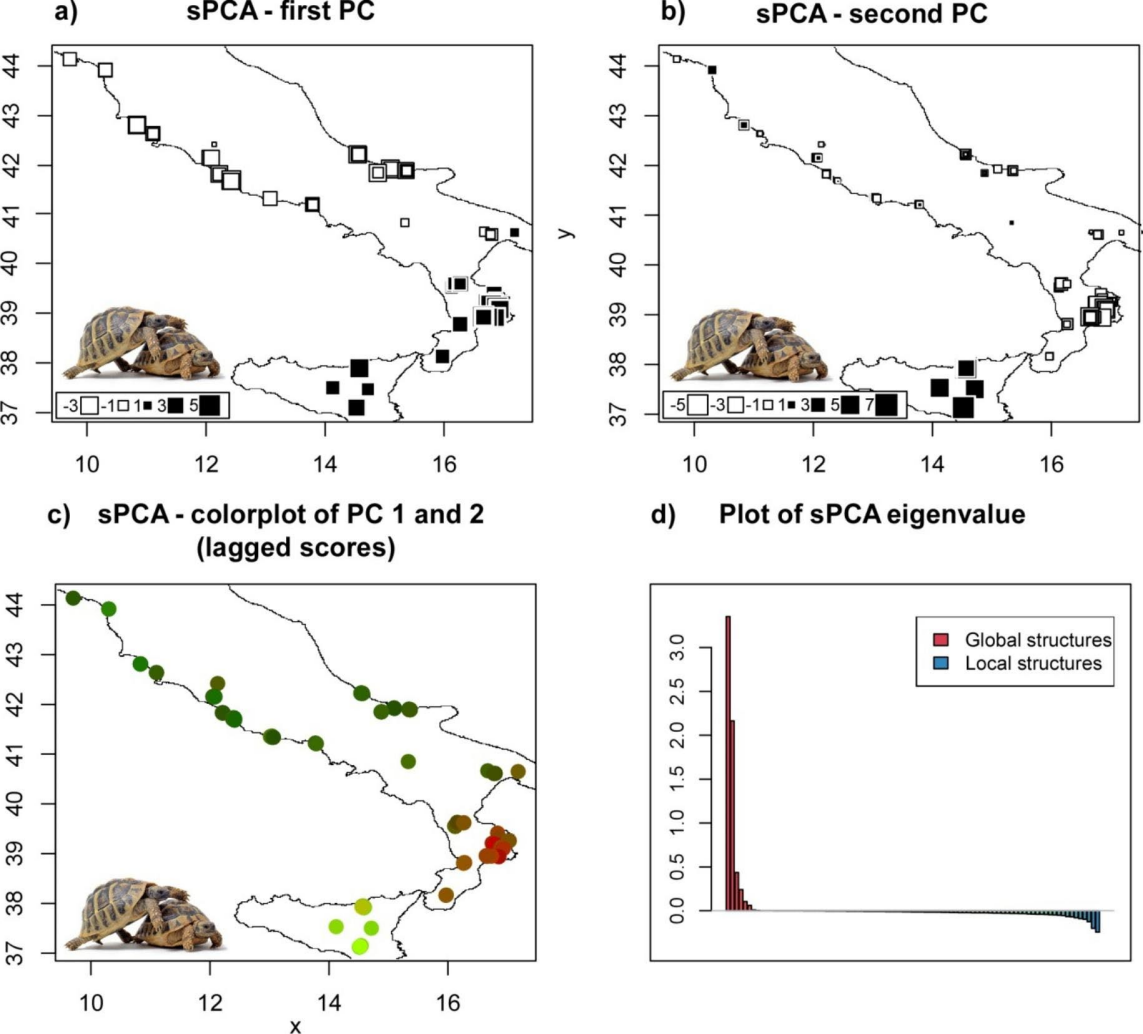
Results from the spatial principal component analysis (sPCA) on the Hermann’s tortoise *Testudo hermanni* in the Italian Peninsula. (a-b) projection of the individual scores from the first two principal components on a distribution map; the color of the boxes (black or white) corresponds to the sign of the score and the area is proportional to the absolute value of the score; therefore large circles indicate large differentiation with smaller circles indicating smaller differences; (c) lagged scores of the first two axes together in a single colour plot, so that individuals that are closely related in the multivariate space are more similar in colour; (d) barplot showing eigenvalues, positive eigenvalues (red, on the left) correspond to global structures, while negative eigenvalues (blues, on the right) indicate local patterns

The spatial Principal Component Analysis (sPCA) performed by the *adegenet* R package [36] revealed a significant geographic structure in the analysed populations. The global permutation test on the eigenvalues showed significant global structure (max(t) = 0.038, P < 0.001), but did not show any significant local structure (max(t) = 0.0189, P = 0.131). The scree plots of the eigenvectors suggest that the first 2 global axes were the most informative axes and were thus retained. For each sample, the scores of the first two principal components were plotted on a distribution map (Fig. 1). The first PC identify two groups, one ranging from Sicily to the north of Calabrian region, and one ranging from the north of Calabrian region to the northern peninsula. The second PC separated the Sicilian samples from the Calabrian samples. Last, we plotted the lagged scores of the first two axes together in a single colour plot, so that individuals that are closely related in the multivariate space are more similar in colour. With this approach, the three main clusters are clearly distinguished on map (Fig. 1c).

**Fig. 2:**
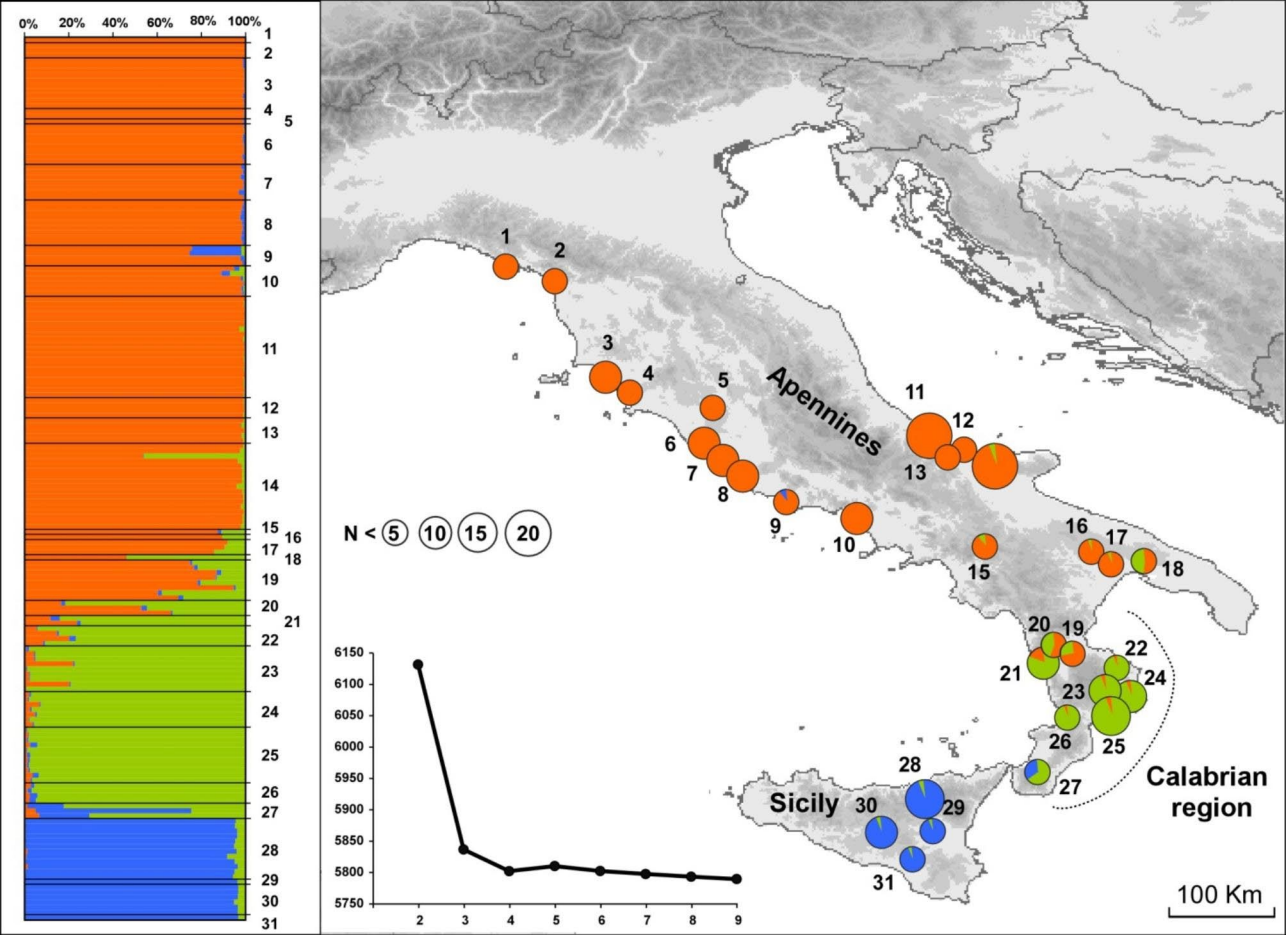
Genetic structure of Italian populations of *Testudo hermanni* at 7 microsatellite loci estimated using TESS. The bar plot on the left shows the admixture proportions of each individual for the three genetic clusters recovered; the pie diagrams on the maps show the frequency distributions of each cluster among the populations; circle size is proportional to the sample size; the line chart shows the mean values of the DIC statistics (averaged over 100 runs) for the number of genetic clusters (K) ranging from 2 to 9. The map was drawn using the software Canvas 11 (ACD Systems of America, Inc.)

The Bayesian clustering analyses carried out with TESS 2.3.1 [37, 38] showed a geographic structuring of genetic variation consistent with results from the sPCA. The plots of the DIC values (i.e. deviance information criterion) versus K values (i.e. the number of clusters) reached a plateau at K = 3 and only a minor decrease in the DIC values was observed at higher K values. Furthermore, the inspection of the plotted membership coefficients for higher K values (see Additional File 1) showed that only three meaningful clusters were represented [39]. Bar-plots showing the individual admixture proportions and pie-charts showing the average proportion of each cluster within each sampled population are given in Fig. 2. The spatial distribution of the three clusters showed a clear geographical structure: one cluster extends in Sicily, one cluster ranges from the south to the north of Calabria, and the third cluster ranges from the north of Calabria to the rest of the peninsula. Large genetic admixture is observed in individuals from the north of Calabria (pop. 19, 20 and 21), as well as in those from the south of Calabrian region (pop. 27).

**Fig. 3:**
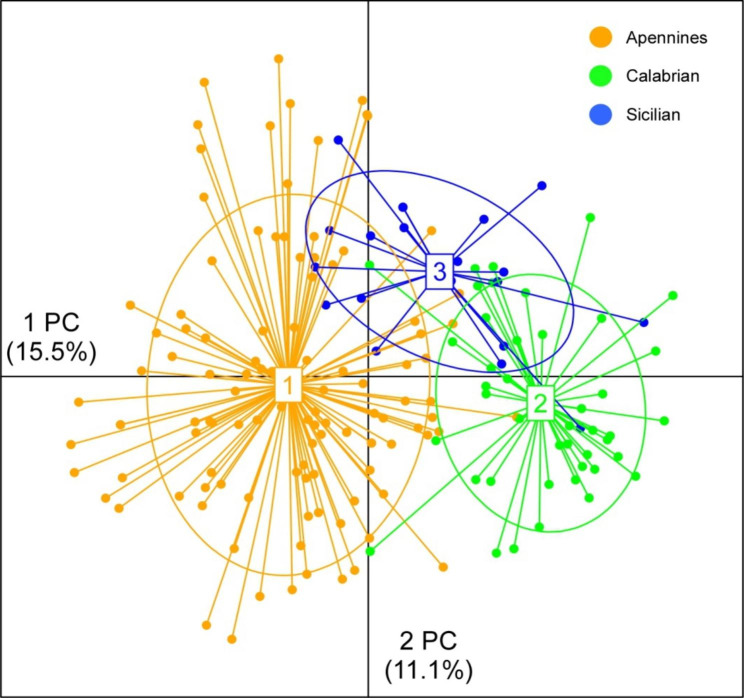
Principal Component Analysis (PCA) on individual genotypes for 7 microsatellites loci. Dots represent individuals, lines connect individuals from a same group. Colors represent the different clusters identified by the TESS analysis (cf. Figure 2)

Finally, results from the standard Principal Component Analysis (PCA) performed on individual genotypes showed a shallow but clear individual clustering in three main groups, which are consistent with the results from previous analyses (see Fig. 3). Indeed, the PC1 allows to easily separate northern from southern individuals, while PC2 show a shallow demarcation between Sicilian and Calabrian individuals.

The analysis of molecular variance (AMOVA) in ARLEQUIN 3.5.1.3 [40] was performed on the groupings resulting from the previous clustering analyses: Apennine group, pop. 1–19; Calabrian group, pop. 20–27; Sicilian group, pop. 28–31. With this grouping, 14.36% of variation had been attributed to the among-group level (FCT: 0.14), 6.57% to the among-population within groups level (FSC: 0.08), whereas the most of variation was attributed to the within population level (79.05%, FST: 0.20); all variance components and fixation indices were statistically significant (P < 0.001). The genetic differentiation among the three clusters, expressed as pairwise Fst estimation, was of the same magnitude, spanning from 0.153 to 0.155 (population pairwise Fst estimates are resumed in the Additional File 2). Estimates of genetic diversity for the three genetic clusters were as follow: Apennine cluster, Ar: 8.9, He: 0.56 (sd 0.21), Ho: 0.46 (sd 0.16); Calabrian cluster: Ar: 8.0, He: 0.59 (sd. 0.24), Ho: 0.54 (0.25); Sicilian cluster: Ar: 5.4, He 0.56 (sd 0.31) Ho 0.51 (sd 0.32).

## Discussion

The analysis of genetic variation of *Testudo hermanni* in the Italian Peninsula and Sicily showed the existence of three main genetic clusters. Previous studies identified some genetic differentiation between populations from the Italian Peninsula and Sicily, and among populations within the peninsula [30, 32]. However, the analysis of the geographic patterns of genetic variation applied here allowed us to identify marked sub-structuring within the southernmost region of the Italian Peninsula, and to define the geographic distributions of the distinct genetic lineages. In fact, we identified one genetic cluster spread in Sicily, one cluster spread from the Aspromonte massif to the Sila mountain chain, and one cluster spread throughout the rest of the Italian Peninsula. Furthermore, we identified two restricted areas of genetic admixture, one in the south of the Aspromonte massif, and one in the north of Calabria, corresponding to the Pollino massif. Overall, our results unveil a hotspot of genetic diversity for *Testudo hermanni* in southern Italy, in an area spanning from the Pollino massif to the Aspromonte massif, and suggest that the interplay between high topographic complexity and Pleistocene climate changes in this region triggered the formation of this hotspot.

The occurrence of genetic sub-structuring and distinct genetic clusters within the Italian Peninsula has been observed in several temperate species including amphibians [16, 17, 22, 23, 25], mammals [14, 18, 20, 41] and reptiles [21], among others [12]. In most of these species, the Calabrian region was identified as a hotspot of genetic lineages [16, 20–23]. Within this region, major mountain areas are arranged along the north-south axis and are separated by lowland fluvial valleys. This topographic structure led glacio-eustatic sea-level oscillations to turn mountain massifs into paleo-islands [42–47]. In particular, these dynamics repeatedly insularized the Sila and Aspromonte massifs, heavily affecting the population structure of terrestrial animal species inhabiting these areas, and leaving detectable imprints in their current genetic structure [14, 16, 22, 23]. This scenario is concordant also with the genetic structure that we identified for the Hermann’s tortoise in southern Italy. Indeed, looking at the distribution of the three genetic clusters, it is possible to identify at least three putative areas acting as Pleistocene refugia for Hermann’s tortoise populations: one located in the southern part of the Calabrian region, one located somewhere north of this area, and one located in Sicily. In the absence of molecular dating analyses, our hypothesis on the Pleistocene history Hermann’s tortoise populations in southern Italy should be taken with caution. However, support for this hypothesis comes also from the fossil record, which identified sites where populations survived during the Late Pleistocene to be located mainly in the southern part of the peninsula, between the Campania and the Calabrian regions [48]. Further investigations involving a higher number of markers (e.g. SNPs and/or nuclear sequence markers) could shed light on the level of divergence among populations as well as on the demographic and evolutionary histories of the three lineages, opening for studies on local adaptation.

Our data clearly identified two areas of genetic admixture, one located in the northern and one in the southern edge of the Calabrian region. These areas, likely originating from secondary contacts among distinct lineages, closely match with areas of secondary contact and admixture observed in several other taxa [14, 22, 23, 49]. Within these areas, gene flow between differentiated lineages boosted the level of population genetic diversity, leading to the comparatively high values of both heterozygosity and allelic richness observed (see Table 1). As a consequence, the whole Calabrian region emerge as a structured hotspot of intraspecific genetic variation for the Hermann’s tortoise, where both unique lineages and high levels of population genetic diversity are found.

Our results have remarkable implications for the management of the Hermann’s tortoise populations. Because of the widespread population decline, *T. hermanni* is considered as *Near Threatened* by the IUCN red list of threatened species at a global scale [50] and as *Endangered* in Italy [51]. However, we identified three unique evolutionarily significant units [52], two of them with narrow and endemic ranges. Assessments of their demographic consistence, as well as of the current threats to their populations have to be planned in the near future, in order to integrate the genetic information into the regional strategies for biodiversity conservation. Furthermore, identifying a hotspot of intraspecific genetic variation in a previously under-investigated region claims for a more detailed investigation on the status of populations inhabiting the hotspot, which might represent a valuable resource for the conservation and management of this species [5]. Indeed, intraspecific genetic variation provides populations with the potential to adapt to the ongoing changes in their biotic and abiotic environment [53, 54]. At the same time, because of the link between genetic diversity and effective population size, these populations are less likely to be affected by the detrimental consequences of genetic drift and inbreeding depression [5, 55–57]. Thus, Hermann’s tortoise populations from the southern part of the Italian Peninsula clearly represent a conservation priority for this species. Finally, the sharp genetic structure identified here, which define the proper geographic distribution of the distinct management units, provides valid support for more informed relocation programs of confiscated animals in the wild [30].

## Conclusion

This study emphasizes the importance of integrating a multi-marker approach with a thorough sampling scheme in investigating the geographic structure of intraspecific genetic variation, in order to unveil hidden patterns of fine-scale genetic structuring. By applying this approach, we identified sharp genetic structure in the Hermann’s tortoise populations from southern Italy. Still, our results add further evidence to the role of Calabrian region as a hotspot of biodiversity, and claim for an assessment of the population genetic structure in other supposed well-known taxa inhabiting this region, with special attention on threatened taxa.

## Methods

We investigated the geographic structure of genetic variation of *Testudo h. hermanni* within the Italian Peninsula and Sicily, by genotyping 174 wild individuals at seven microsatellite loci (*Test10*, *Test56*, *Test71*, *Test76*, *Gal136*, *Gal75*, and *Gal263*), following the protocols described in Biello et al. [30].

Collecting sites and sample sizes are given in Table 1. Samples were collected from 2010 to 2018, mainly from March to June and from September to October, i.e. the period of maximum species activity. Considering the low dispersal capacity of this species [58], and the fragmentation of the species habitat in Italy [59], each collection site can be considered a distinct breeding population. Genomic DNA was obtained from blood samples collected from the nape or coccygeal vein. About 75 µL of blood were spotted on FTA® Classic Cards (Whatman™, GE Healthcare) and stored at room temperature. Alternatively, whole blood samples (100 µL – 1 mL) were treated with K3-EDTA and stored at -20 °C. DNA was extracted from both FTA-Cards and whole blood samples using a suspension of 5% Chelex® 100 Resin. Field works, collection of tissues, and the experimental protocols were performed in accordance with the relevant guidelines and regulations (including ethics guidelines and regulations), and were approved by the Italian Ministry of Environment, Land and Sea Protection (permit codes: 0044068–4/12/2012-PNM-II; 0001805/PNM − 4/2/2015; ISPRA 68,754/T-A31–28/11/2016).

Fragment analysis of PCR products was performed by Macrogen Inc. on an ABI 3730xl Genetic Analyser (Applied Biosystems) with a 400HD size standard. Electropherograms checking and allele calling was performed by using GENEMAPPER® 4.1. Micro-Checker 2.2.3 [34] was used to test for null alleles and large-allele dropout influences. Allelic frequencies were then computed by using GENETIX 4.05 [60], while FSTAT [35] was used to test for deviations from the expected Hardy-Weinberg and linkage equilibria, as well as to estimate population pairwise Fst values. We also estimated the mean allelic richness and the mean observed and expected (unbiased) heterozygosity using the *adegenet* package in R environment [36]; allelic richness was computed using the rarefaction method [61].

The extent of spatial patterns of genetic variation was investigated by a spatial Principal Components Analysis (sPCA), as implemented in the *adegenet* R package [36]. The analysis takes into account the spatial autocorrelation (Moran’s I) of genetic data to estimate coefficients of similarity among individuals. Jittered geographic coordinates of sampling location for each individual were used to build two different networks, a Delaunay triangulation and a Neighbourhood by distance network. Since no significant differences were observed in preliminary results, we only retained the Neighbourhood by distance network, which best represents realistic connections among the sampled populations (i.e. closer populations are more likely to be connected than farther populations). The significance of global (neighbouring individuals are more similar than expected) and local (neighbouring individuals are more dissimilar than expected) structures of genetic variation was assessed with a Monte Carlo-based test (9999 iterations). Results from the first two principal components were mapped in geographic space and examined to cluster samples into genetically and geographically distinct populations.

The genetic clustering of individuals across the study area was also investigated using the Bayesian clustering algorithm implemented in TESS 2.3.1 and the geographical location of individuals as prior information [37, 38]. The analysis was performed by modelling admixture using a conditional autoregressive model (CAR). Preliminary analyses were carried out to assess model performance, with 20 000 steps (the first 5 000 were discarded as burn-in) and 10 replicates for each K value (i.e. the number of clusters) between 2 and 31. The final analysis contained 100 replicates for each K value, with K = 2–9; each run consisted of 80 000 steps, with the first 30 000 discarded as burn-in. The spatial interaction parameter was initially kept at the default value (0.6), and the updating option was activated. The model that best fitted the data was selected using the deviance information criterion (DIC). DIC values were averaged over the 100 replicates for each K value, and the most probable K value was selected as the one at which the average DIC reached a plateau. For the selected K value, the estimated admixture proportions of the 10 runs with the lowest DIC were averaged using CLUMPP 1.1.2 [62].

Finally, the genetic clustering of individuals was also evaluated without accounting for spatial information by means of a standard PCA of genotypic data, as implemented in the *adegenet* R package [36].

In order to estimate the amount of variation attributable to differences among the clusters identified by the clustering methods, among populations within clusters, and within populations, a locus by locus analysis of molecular variance (AMOVA) was performed using ARLEQUIN 3.5.1.3 [40]. Populations resulting as admixed were attributed to the most represented cluster. The significance of variance components and fixation indices was tested using 1092 permutations. Using this grouping, we estimated the pairwise Fst among group, the mean allelic richness, and the mean observed and expected (unbiased) heterozygosity within the main clusters using the *adegenet* R package.

## Electronic supplementary material

Below is the link to the electronic supplementary material.

Supplementary Material 1

Supplementary Material 2

## Data Availability

The datasets generated and/or analysed during the current study are available in the ZENODO repository at the following link: 10.5281/zenodo.6566199.
