# Supplementary material for: Unveiling a hotspot of genetic diversity in southern Italy for the endangered Hermann’s tortoise Testudo hermanni
Source: BMC Ecol Evol. 2022 Nov 7;22:131. doi: 10.1186/s12862-022-02075-w (PMC9641751; doi:10.1186/s12862-022-02075-w)
Supplement: Supplementary file 1 — Supplementary Material 1 [file 12862_2022_2075_MOESM1_ESM.pdf]

Supplement to: Unveiling a hotspot of genetic diversity in southern Italy for the endangered Hermann's tortoise Testudo hermanni

Additional file 2 – **Supplementary results: Population pairwise Fst estimates, and Confidence interval estimated by means of 1000 bootstrapping replicates as implemented in the "hierfstat" R package** (Goudet et al., 2005, doi.org/10.1111/j.1471-8286.2004.00828.x); populations are numbered as in the main text' Table 1.

Supplementary Table 1 - Population pairwise Fst

	1	2	3	4	5	6	7	8	9	10	11	12	13	14	15	16	17	18	19	20	21	22	23	24	25	26	27	28	29	30	31
**1**	-	0.090	-0.035	-0.190	0.000	0.026	0.013	0.040	0.215	0.137	-0.005	0.136	0.200	0.007	0.000	0.000	0.248	0.000	0.010	-0.096	0.326	0.232	0.212	0.250	0.363	0.402	0.396	0.293	0.000	0.276	0.000
**2**		-	0.022	-0.004	0.143	0.033	0.038	0.028	0.085	0.123	0.023	0.065	0.085	0.003	0.236	0.316	0.122	0.348	0.133	0.117	0.316	0.178	0.217	0.206	0.292	0.321	0.220	0.169	0.182	0.107	0.270
**3**			-	0.022	0.047	0.003	0.112	0.082	0.146	0.183	0.060	0.005	0.086	0.104	0.047	0.061	0.118	0.276	0.128	0.178	0.270	0.236	0.286	0.245	0.362	0.338	0.276	0.239	0.129	0.191	0.145
**4**				-	-0.101	0.006	-0.114	-0.041	0.004	0.046	-0.011	0.017	0.101	-0.088	0.109	0.189	0.117	0.065	-0.040	-0.083	0.163	0.040	0.122	0.144	0.237	0.265	0.179	0.157	0.060	0.178	0.113
**5**					-	-0.048	-0.050	-0.039	-0.079	0.050	-0.057	0.230	0.084	-0.040	0.000	0.000	0.175	0.000	0.091	-0.269	0.287	0.103	0.004	-0.047	0.150	0.189	0.103	0.019	0.000	0.093	0.000
**6**						-	0.042	0.024	0.077	0.098	0.017	0.009	0.074	0.050	-0.012	0.042	0.056	0.171	0.076	0.081	0.221	0.169	0.202	0.155	0.263	0.241	0.164	0.185	0.132	0.156	0.164
**7**							-	-0.027	-0.008	0.059	0.042	0.028	0.110	-0.024	0.026	0.184	0.087	0.074	0.046	0.019	0.161	0.086	0.149	0.153	0.202	0.203	0.089	0.177	0.138	0.189	0.190
**8**								-	0.006	0.109	0.064	0.061	0.127	-0.014	0.007	0.109	0.118	0.133	0.075	0.078	0.207	0.115	0.183	0.152	0.241	0.233	0.114	0.170	0.124	0.170	0.185
**9**									-	0.123	0.062	0.113	0.032	-0.002	-0.142	0.251	0.093	0.110	0.173	-0.001	0.279	0.123	0.126	0.131	0.202	0.192	0.041	0.111	0.118	0.127	0.265
**10**										-	0.007	0.112	0.134	0.086	0.257	0.447	0.049	0.198	0.156	0.057	0.331	0.221	0.191	0.217	0.211	0.237	0.138	0.248	0.300	0.236	0.384
**11**											-	-0.020	0.014	0.066	0.021	0.227	-0.007	0.163	0.124	0.073	0.265	0.197	0.203	0.201	0.252	0.235	0.154	0.213	0.159	0.185	0.224
**12**												-	0.063	0.046	0.241	0.371	0.112	0.393	0.113	0.127	0.345	0.232	0.222	0.225	0.315	0.333	0.300	0.252	0.332	0.243	0.364
**13**													-	0.109	-0.048	0.310	-0.024	0.248	0.237	0.106	0.366	0.276	0.242	0.247	0.312	0.319	0.217	0.236	0.220	0.188	0.319
**14**														-	0.050	0.179	0.095	0.130	0.090	0.064	0.193	0.106	0.182	0.180	0.231	0.224	0.105	0.171	0.093	0.161	0.183
**15**															-	0.000	0.111	0.000	0.181	-0.103	0.322	0.197	0.108	0.055	0.215	0.189	0.146	0.090	0.000	0.145	0.000
**16**																-	0.403	0.000	0.138	0.125	0.267	0.284	0.311	0.199	0.407	0.445	0.396	0.232	0.000	0.268	0.000
**17**																	-	0.283	0.206	0.055	0.308	0.260	0.202	0.211	0.221	0.267	0.184	0.245	0.327	0.187	0.398
**18**																		-	0.151	-0.147	0.303	0.163	0.129	0.119	0.116	0.160	0.032	0.157	0.000	0.217	0.000
**19**																			-	0.065	0.044	0.099	0.187	0.176	0.247	0.242	0.197	0.204	0.199	0.239	0.159
**20**																				-	0.066	0.056	-0.001	0.040	0.074	0.058	-0.010	0.133	0.000	0.139	0.087
**21**																					-	0.151	0.190	0.163	0.220	0.340	0.294	0.231	0.114	0.286	0.103
**22**																						-	0.044	0.070	0.101	0.099	0.131	0.120	0.222	0.206	0.232
**23**																							-	0.028	0.072	0.056	0.117	0.172	0.217	0.228	0.262
**24**																								-	0.092	0.096	0.113	0.140	0.170	0.193	0.202
**25**																									-	0.014	0.060	0.229	0.314	0.258	0.370
**26**																										-	0.069	0.202	0.363	0.252	0.417
**27**																											-	0.126	0.264	0.131	0.374
**28**																												-	-0.006	0.066	0.063
**29**																													-	-0.048	0.000
**30**																														-	0.164
**31**																															-

Supplementary Table 2 - bootstrapping over loci of pairwise Fst: Upper limit above the diagonal, lower limit below the diagonal.

	1	2	3	4	5	6	7	8	9	10	11	12	13	14	15	16	17	18	19	20	21	22	23	24	25	26	27	28	29	30	31
**1**	-	0.346	0.223	0.134	0.000	0.205	0.194	0.189	0.581	0.440	0.187	0.247	0.458	0.163	0.000	0.000	0.481	0.000	0.178	0.260	0.503	0.390	0.513	0.439	0.615	0.758	0.684	0.417	0.000	0.411	0.000
**2**	-0.112	-	0.083	0.126	0.391	0.122	0.111	0.095	0.273	0.248	0.064	0.168	0.141	0.033	0.559	0.581	0.169	0.601	0.268	0.279	0.564	0.359	0.394	0.344	0.465	0.633	0.367	0.240	0.270	0.144	0.421
**3**	-0.365	-0.052	-	0.138	0.253	0.038	0.226	0.165	0.336	0.353	0.115	0.060	0.194	0.160	0.260	0.190	0.224	0.396	0.234	0.276	0.362	0.285	0.381	0.320	0.500	0.462	0.398	0.281	0.299	0.250	0.302
**4**	-0.520	-0.109	-0.087	-	0.152	0.137	-0.053	0.038	0.140	0.162	0.097	0.110	0.254	-0.053	0.353	0.449	0.279	0.340	0.006	0.045	0.326	0.160	0.222	0.236	0.327	0.425	0.324	0.244	0.359	0.286	0.358
**5**	0.000	-0.067	-0.135	-0.441	-	0.038	0.006	0.035	0.040	0.222	0.082	0.399	0.264	0.096	0.000	0.000	0.427	0.000	0.314	-0.055	0.417	0.194	0.059	0.054	0.300	0.457	0.167	0.126	0.000	0.176	0.000
**6**	-0.152	-0.049	-0.035	-0.113	-0.153	-	0.145	0.069	0.178	0.274	0.057	0.048	0.160	0.095	0.108	0.121	0.167	0.312	0.165	0.197	0.332	0.222	0.277	0.253	0.399	0.352	0.270	0.238	0.292	0.235	0.305
**7**	-0.209	-0.038	-0.008	-0.204	-0.123	-0.033	-	0.015	0.057	0.148	0.103	0.069	0.219	0.000	0.190	0.387	0.171	0.223	0.108	0.082	0.276	0.155	0.181	0.216	0.249	0.306	0.153	0.278	0.305	0.303	0.342
**8**	-0.130	-0.036	-0.007	-0.124	-0.215	-0.019	-0.063	-	0.092	0.214	0.134	0.138	0.206	0.011	0.131	0.231	0.185	0.239	0.126	0.115	0.285	0.200	0.242	0.231	0.284	0.362	0.162	0.228	0.260	0.234	0.286
**9**	-0.179	-0.034	-0.058	-0.138	-0.286	-0.015	-0.057	-0.048	-	0.273	0.149	0.278	0.074	0.070	0.043	0.607	0.212	0.267	0.387	0.047	0.479	0.175	0.152	0.204	0.304	0.297	0.106	0.168	0.308	0.186	0.607
**10**	-0.233	0.022	-0.009	-0.059	-0.092	-0.027	-0.030	0.005	-0.018	-	0.088	0.208	0.371	0.137	0.525	0.682	0.207	0.387	0.290	0.120	0.418	0.286	0.249	0.287	0.274	0.404	0.246	0.373	0.556	0.407	0.621
**11**	-0.203	-0.015	-0.005	-0.100	-0.175	-0.015	-0.023	-0.007	-0.042	-0.042	-	0.010	0.064	0.123	0.152	0.303	0.069	0.316	0.184	0.115	0.373	0.268	0.250	0.263	0.295	0.350	0.228	0.280	0.309	0.266	0.354
**12**	0.051	-0.003	-0.052	-0.079	0.013	-0.033	-0.027	-0.033	-0.050	0.032	-0.053	-	0.104	0.109	0.381	0.464	0.188	0.529	0.169	0.197	0.445	0.314	0.313	0.283	0.421	0.483	0.392	0.344	0.490	0.305	0.512
**13**	-0.149	0.038	-0.025	-0.062	-0.183	-0.003	0.012	0.033	-0.014	-0.040	-0.042	0.012	-	0.160	0.032	0.510	0.089	0.376	0.357	0.267	0.472	0.365	0.322	0.332	0.492	0.452	0.374	0.324	0.351	0.298	0.469
**14**	-0.164	-0.030	0.038	-0.135	-0.143	0.007	-0.043	-0.034	-0.037	0.022	0.001	-0.027	-0.003	-	0.214	0.321	0.185	0.238	0.182	0.163	0.297	0.211	0.262	0.295	0.276	0.369	0.210	0.240	0.204	0.244	0.267
**15**	0.000	-0.008	-0.320	-0.259	0.000	-0.153	-0.136	-0.137	-0.360	-0.067	-0.171	-0.056	-0.157	-0.126	-	0.000	0.301	0.000	0.366	0.223	0.481	0.343	0.250	0.202	0.526	0.419	0.418	0.154	0.000	0.227	0.000
**16**	0.000	0.101	-0.101	-0.265	0.000	-0.072	0.012	-0.005	-0.010	0.192	0.074	0.252	0.091	0.016	0.000	-	0.650	0.000	0.344	0.477	0.455	0.465	0.541	0.421	0.713	0.750	0.728	0.344	0.000	0.399	0.000
**17**	-0.115	0.091	-0.035	-0.128	-0.323	-0.063	-0.061	0.005	-0.080	-0.106	-0.087	0.023	-0.191	-0.028	-0.263	0.054	-	0.527	0.314	0.168	0.481	0.326	0.273	0.290	0.332	0.406	0.306	0.356	0.535	0.282	0.599
**18**	0.000	0.172	0.072	-0.458	0.000	-0.028	-0.153	-0.002	-0.136	-0.083	-0.062	0.198	0.029	-0.058	0.000	0.000	-0.254	-	0.331	0.000	0.503	0.331	0.225	0.230	0.178	0.455	0.145	0.287	0.000	0.350	0.000
**19**	-0.169	0.020	0.057	-0.091	-0.083	0.017	-0.006	0.030	0.029	0.057	0.069	0.052	0.143	0.019	-0.028	-0.037	0.095	-0.104	-	0.220	0.157	0.148	0.363	0.299	0.446	0.423	0.397	0.292	0.333	0.322	0.319
**20**	-0.480	0.011	0.068	-0.194	-0.453	-0.014	-0.064	0.027	-0.068	-0.018	0.022	0.041	-0.070	-0.028	-0.342	-0.192	-0.110	-0.487	-0.030	-	0.170	0.139	0.046	0.115	0.137	0.199	0.040	0.290	0.302	0.308	0.370
**21**	0.043	0.099	0.102	-0.120	0.125	0.072	-0.015	0.089	0.064	0.155	0.124	0.204	0.213	0.062	0.135	-0.033	0.038	0.021	-0.114	-0.119	-	0.275	0.360	0.296	0.465	0.603	0.516	0.315	0.292	0.414	0.285
**22**	0.094	0.049	0.175	-0.109	-0.027	0.090	-0.026	0.015	0.075	0.091	0.086	0.122	0.186	-0.003	0.038	0.116	0.168	-0.059	0.007	-0.020	-0.037	-	0.104	0.164	0.213	0.232	0.314	0.257	0.369	0.329	0.363
**23**	-0.072	0.092	0.221	0.043	-0.079	0.141	0.103	0.132	0.106	0.122	0.140	0.144	0.152	0.093	-0.094	0.087	0.123	-0.066	0.086	-0.052	0.034	-0.012	-	0.090	0.121	0.119	0.211	0.248	0.341	0.300	0.487
**24**	0.111	0.069	0.166	0.070	-0.286	0.039	0.102	0.040	0.077	0.137	0.130	0.141	0.155	0.069	-0.084	-0.011	0.117	0.013	0.075	-0.028	0.032	-0.003	-0.007	-	0.229	0.158	0.235	0.252	0.239	0.293	0.412
**25**	0.091	0.166	0.235	0.164	0.004	0.173	0.130	0.167	0.123	0.120	0.172	0.193	0.190	0.173	-0.032	0.091	0.147	0.020	0.102	-0.003	0.016	0.006	0.029	0.018	-	0.071	0.090	0.405	0.577	0.450	0.675
**26**	0.096	0.114	0.219	0.103	0.013	0.143	0.095	0.134	0.138	0.081	0.134	0.225	0.166	0.087	0.000	0.136	0.158	-0.135	0.096	-0.043	0.100	-0.028	-0.014	0.047	-0.035	-	0.196	0.325	0.645	0.422	0.731
**27**	0.136	0.132	0.126	0.017	0.028	0.060	0.012	0.068	-0.036	0.023	0.043	0.190	0.040	0.032	-0.080	-0.027	-0.014	-0.267	0.032	-0.066	0.021	-0.025	0.042	0.004	0.027	-0.009	-	0.274	0.459	0.302	0.678
**28**	0.146	0.114	0.177	0.051	-0.137	0.110	0.074	0.099	0.045	0.105	0.105	0.143	0.110	0.090	-0.026	0.121	0.087	0.044	0.134	-0.017	0.135	-0.006	0.110	0.060	0.097	0.088	-0.045	-	0.061	0.103	0.251
**29**	0.000	0.082	-0.145	-0.682	0.000	-0.096	-0.111	-0.077	-0.094	-0.018	-0.115	0.120	-0.056	-0.140	0.000	0.000	-0.130	0.000	0.045	-0.309	-0.180	0.022	0.091	0.102	0.090	0.060	0.093	-0.074	-	0.054	0.000
**30**	0.084	0.046	0.104	0.028	0.015	0.061	0.055	0.057	0.048	0.049	0.059	0.131	0.025	0.043	0.050	0.108	0.037	0.036	0.173	0.024	0.166	0.086	0.165	0.106	0.108	0.064	0.002	0.027	-0.244	-	0.302
**31**	0.000	0.189	-0.103	-0.367	0.000	0.000	0.006	0.037	-0.072	0.088	-0.013	0.168	0.091	0.026	0.000	0.000	0.014	0.000	-0.022	-0.250	-0.182	0.107	0.013	0.062	0.044	0.109	0.045	-0.162	0.000	0.024	-
