# Supplementary material for: Unveiling a hotspot of genetic diversity in southern Italy for the endangered Hermann’s tortoise Testudo hermanni
Source: BMC Ecol Evol. 2022 Nov 7;22:131. doi: 10.1186/s12862-022-02075-w (PMC9641751; doi:10.1186/s12862-022-02075-w)

Supplement to: Unveiling a hotspot of genetic diversity in southern Italy for the endangered Hermann's tortoise *Testudo hermanni*

Additional file 1 – Supplementary results from the TESS Analysis.

Bar plots showing the admixture proportions of each individual for the genetic clusters recovered in the Bayesian clustering analysis performed on TESS, setting the number of genetic clusters (K) from 2 to 9; each colour represents a different clusters. Details are provided in the main text.

K = 2

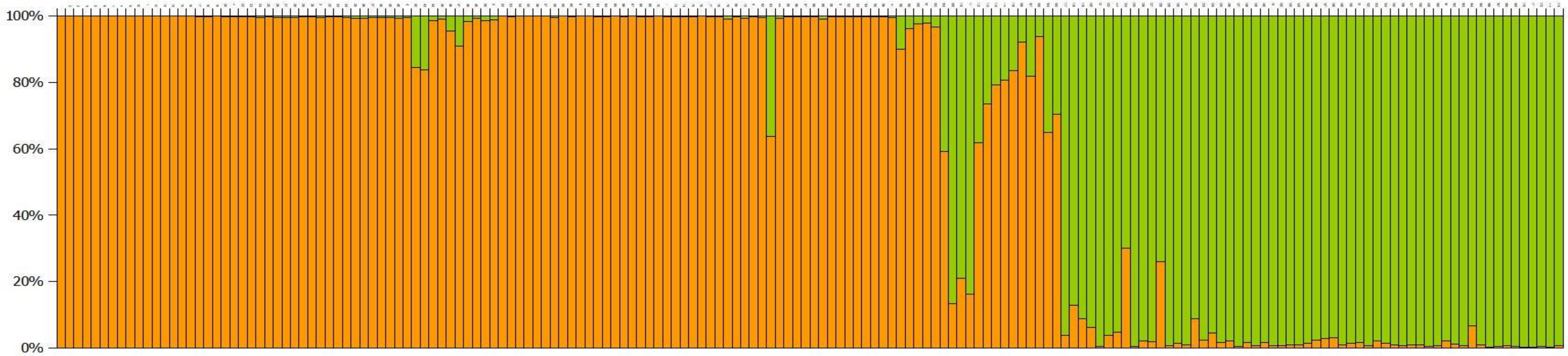

K = 3

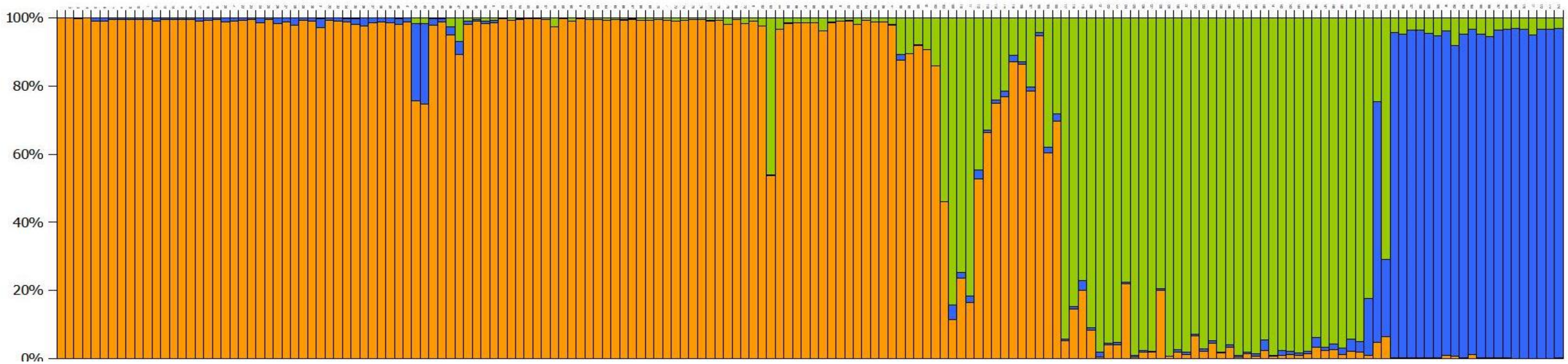

K = 4

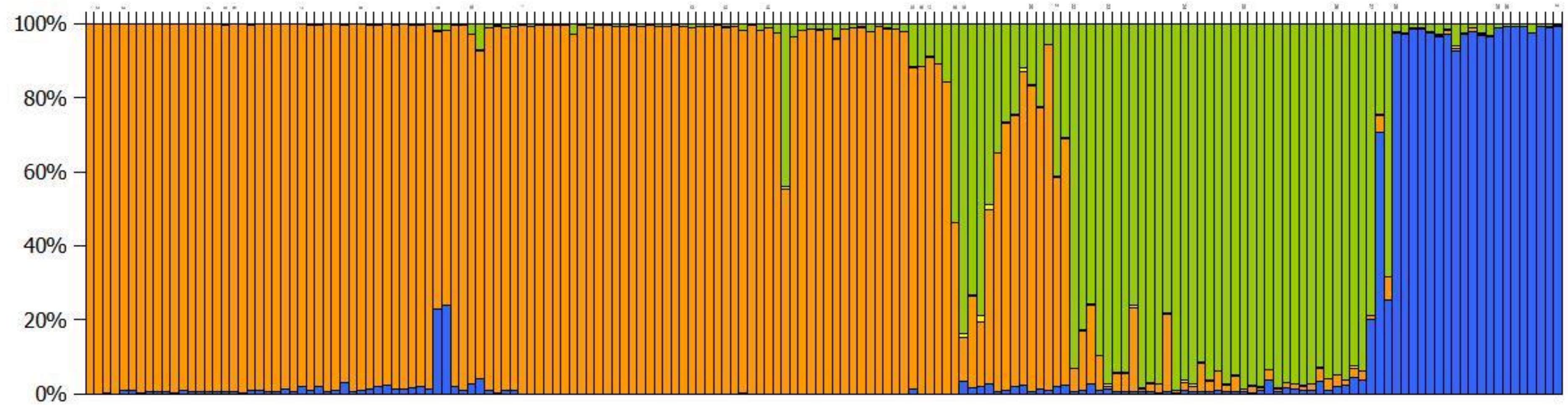

K = 5

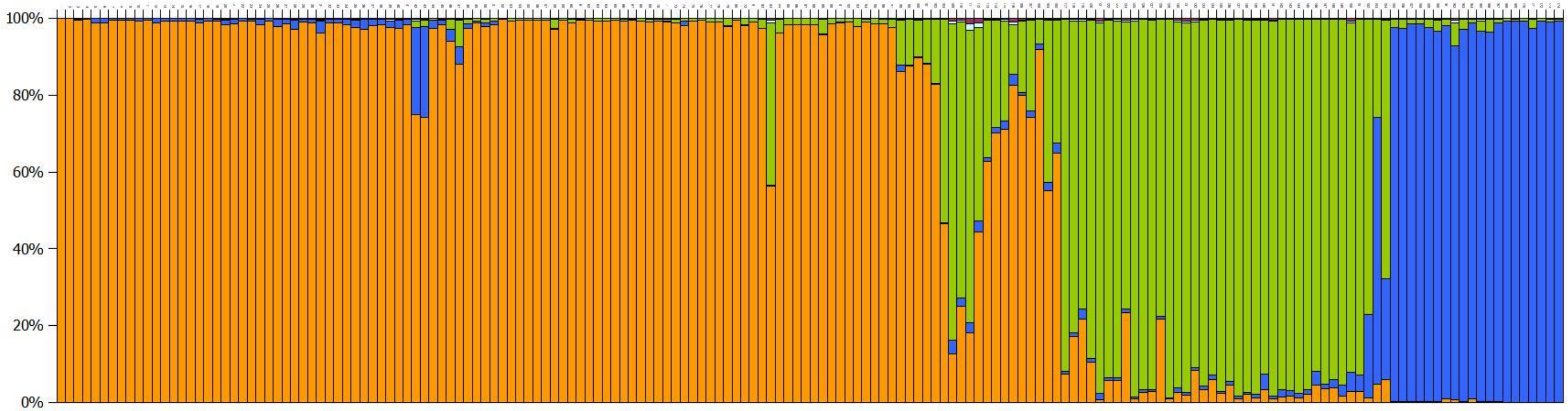

K = 6

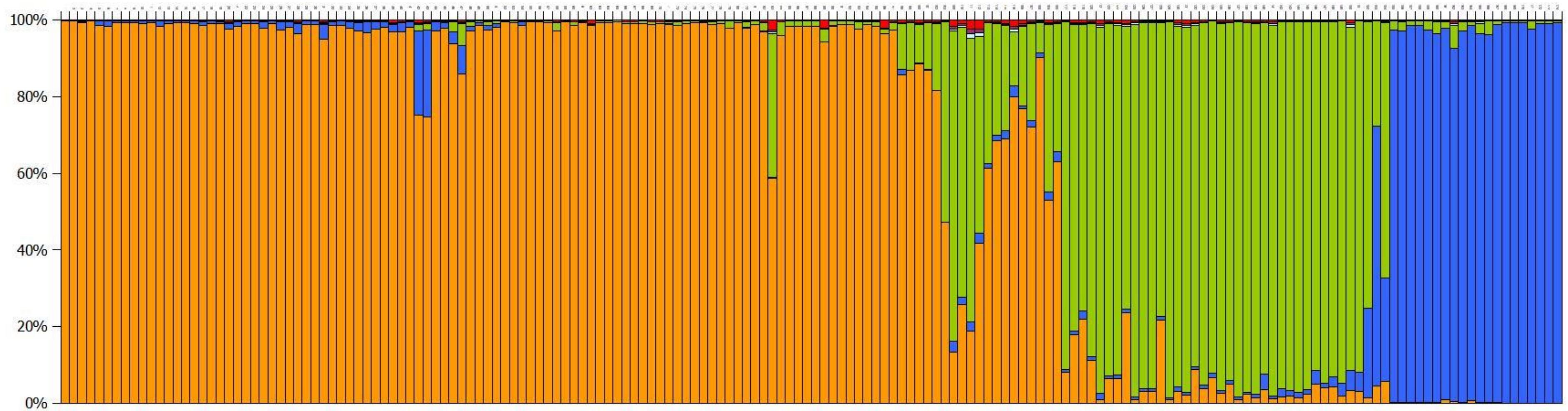

K = 7

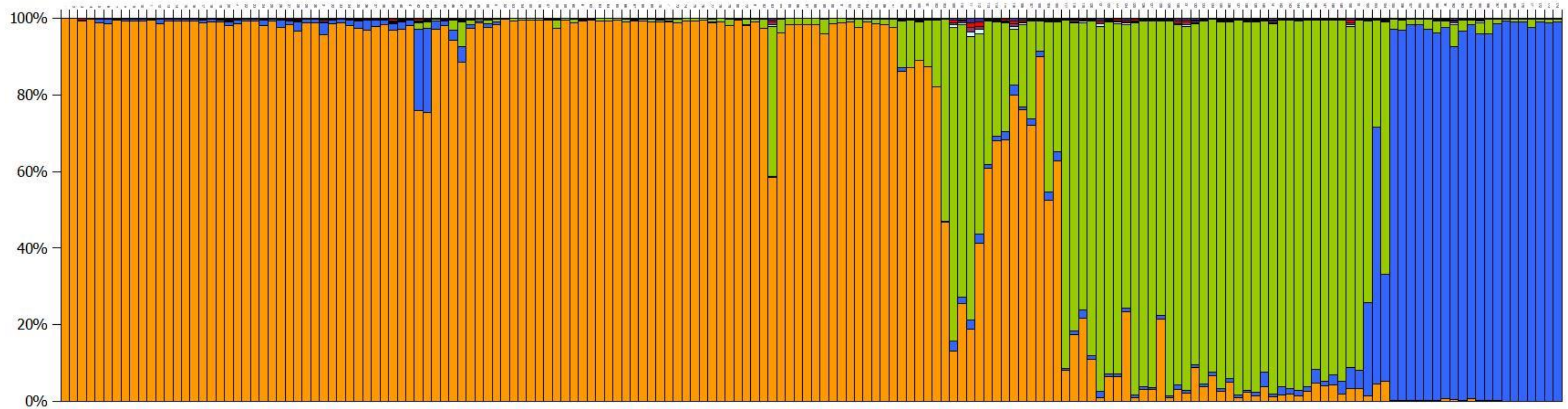

K = 8

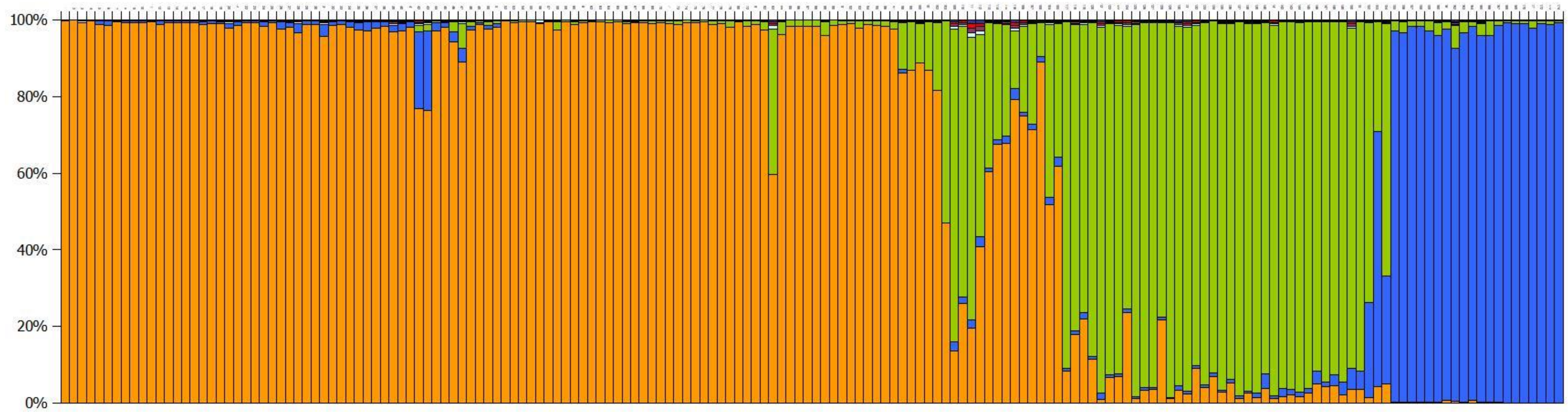

K = 9

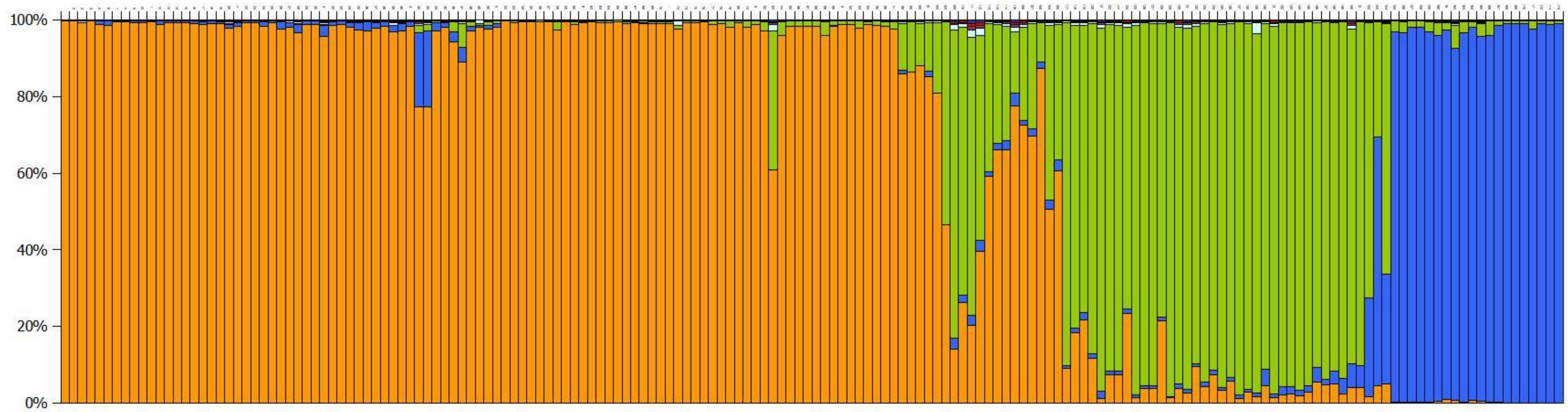

Supplement: Supplementary file 2 — Supplementary Material 2 [file 12862_2022_2075_MOESM2_ESM.pdf]
